# Safety and efficacy of everolimus in Chinese patients with metastatic renal cell carcinoma resistant to vascular endothelial growth factor receptor-tyrosine kinase inhibitor therapy: an open-label phase 1b study

**DOI:** 10.1186/1471-2407-13-136

**Published:** 2013-03-21

**Authors:** Jun Guo, Yiran Huang, Xu Zhang, Fangjian Zhou, Yinghao Sun, Shukui Qin, Zhangqun Ye, Hui Wang, Annette Jappe, Patrick Straub, Nicoletta Pirotta, Sven Gogov

**Affiliations:** 1 Peking University Cancer Hospital and Institute, No. 52, Fucheng Road, Beijing, 100142, China; 2 Shanghai Renji Hospital, Shanghai, China; 3 The General Hospital of PLA, Beijing, China; 4 Sun Yat-sen University Cancer Center, Guangzhou, China; 5 Shanghai Changhai Hospital, Yangpu District, China; 6 Nanjing Bayi Hospital, Yanggongjing, Nanjing, China; 7 Wuhan Tongji Hospital, Wuhan, China; 8 Beijing Novartis Pharma Company, Ltd., Beijing, China; 9 Novartis Pharma AG, Basel, Switzerland; 10 Novartis Pharma AG, Basel, Switzerland; 11 Novartis Pharma AG, Basel, Switzerland; 12 Novartis Pharma AG, Basel, Switzerland

**Keywords:** Asian, Everolimus, mTOR inhibitor, Renal cell cancer, Sunitinib, Sorafenib

## Abstract

**Background:**

In China, there are currently no approved therapies for the treatment of metastatic renal cell carcinoma (mRCC) following progression with vascular endothelial growth factor (VEGF)-targeted agents. In the phase 3 RECORD-1 trial, the mammalian target of rapamycin (mTOR) inhibitor everolimus afforded clinical benefit with good tolerability in Western patients with mRCC whose disease had progressed despite VEGF receptor-tyrosine kinase inhibitor (VEGFr-TKI) therapy. This phase 1b study was designed to further evaluate the safety and efficacy of everolimus in VEGFr-TKI-refractory Chinese patients with mRCC.

**Methods:**

An open-label, multicenter phase 1b study enrolled Chinese patients with mRCC who were intolerant to, or progressed on, previous VEGFr-TKI therapy (N = 64). Patients received everolimus 10 mg daily until objective tumor progression (according to RECIST, version 1.0), unacceptable toxicity, death, or study discontinuation for any other reason. The final data analysis cut-off date was November 30, 2011.

**Results:**

A total of 64 patients were included in the study. Median age was 52 years (range, 19–75 years) and 69% of patients were male. Median duration of everolimus therapy was 4.1 months (range, 0.0-16.1 months). Expected known class-effect toxicities related to mTOR inhibitor therapy were observed, including anemia (64%), hypertriglyceridemia (55%), mouth ulceration (53%), hyperglycemia (52%), hypercholesterolemia (50%), and pulmonary events (31%). Common grade 3/4 adverse events were anemia (20%), hyperglycemia (13%), increased gamma-glutamyltransferase (11%), hyponatremia (8%), dyspnea (8%), hypertriglyceridemia (6%), and lymphopenia (6%). Median PFS was 6.9 months (95% CI, 3.7-12.5 months) and the overall tumor response rate was 5% (95% CI, 1-13%). The majority of patients (61%) had stable disease as their best overall tumor response.

**Conclusions:**

Safety and efficacy results were comparable to those of the RECORD-1 trial. Everolimus is generally well tolerated and provides clinical benefit to Chinese patients with anti-VEGF-refractory mRCC.

**Trial registration:**

clinicaltrials.gov, NCT01152801

## Background

Renal cell carcinoma (RCC), the most common form of kidney tumor, accounts for up to 92% of all cases of kidney cancer [1]. In the United States alone, an estimated 64,770 new cases of renal tumors are expected to be diagnosed during 2012, which will ultimately attribute to 13,570 deaths [1]. The incidence of kidney cancer in China is low, compared with the average global incidence rate (2.1 vs 4.0 per 100,000) [2]. However, the incidence of and death rate from kidney cancer in China has risen during recent years [3].

Surgery forms the primary standard of care for most localized kidney cancers [1]. However, one third of patients who undergo surgery for localized disease will experience recurrence and approximately a quarter of patients have locally invasive or metastatic RCC (mRCC) at the time of diagnosis [4]. In such settings, targeted agents have been shown to afford significant clinical benefit with acceptable safety [5-11]. The vascular endothelial growth factor receptor-tyrosine kinase inhibitors (VEGFr-TKIs) sunitinib and sorafenib are approved in China for first-line treatment of patients with mRCC.

Sequential lines of therapy are typically required to maintain clinical benefit in patients with mRCC. Clinical practice guidelines in the United States and Europe recommend targeted agents or cytokines for first- and subsequent-line treatment of patients with mRCC [12-15]. VEGF-targeted agents are recommended as first-line therapy for the majority of patients (those who are at low or intermediate risk). Everolimus is recommended for patients who fail initial VEGFr-TKI therapy and axitinib for patients who fail previous systemic therapy. Although there are currently no approved therapies in China for the treatment of patients with mRCC refractory to VEGFr-TKIs, local clinical practice guidelines recommend everolimus for this patient population.

The PI3K/AKT/mTOR pathway is dysregulated in many cancers, including RCC [16]. mTOR is a serine/threonine kinase that binds specifically to the FK506 binding protein 12 (FKBP12)-rapamycin complex [17]. mTOR is activated by components of the PI3K pathway and tuberous sclerosis complex (TSC) and regulates protein synthesis required for cell growth and proliferation, metabolism, and angiogenesis [18]. Overactivation of mTOR signaling occurs via a number of mechanisms, including overexpression or activation of growth factor receptors, activating mutations in PI3K/AKT, or decreased expression of TSC [18]. Overproduction of VEGF and other growth factors in tumor cells leads to activation of mTOR signaling in neighboring endothelial cells, thereby increasing angiogenesis [18]. Inhibition of mTOR signaling results in decreased cell growth and proliferation, cellular metabolism, and angiogenesis, ultimately leading to cell cycle block at the G1 phase [19]. The mTOR inhibitor everolimus binds to the intracellular protein FKBP-12, forming a complex that inhibits the mTOR serine-threonine kinase [19].

Everolimus has been evaluated in patients with cancer in multiple clinical studies. Phase 1 PK/PD studies demonstrated that continuous daily dosing with everolimus 10 mg resulted in a more profound and sustained inhibition of mTOR than that achieved with a weekly dosage schedule [20,21]. Anti-tumor activity of everolimus 10 mg daily was shown in a phase 2 trial of patients with mRCC [22,23], and results of the phase 3 RECORD-1 study demonstrated a progression-free survival (PFS) benefit of everolimus 10 mg daily over placebo in patients with VEGFr-TKI–refractory mRCC (4.9 vs 1.9 months; HR 0.33; 95% CI, 0.25-0.43; *P* < .001) [10]. In addition, pharmacodynamic modeling of tumor growth in patients enrolled in RECORD-1 demonstrated that everolimus 5 mg daily and 10 mg daily significantly slowed the growth of mRCC target lesions, non-target lesions, and new metastases compared with placebo (*P* < .001) [24,25].

A phase 1 study in Chinese patients with advanced solid tumors (N = 24), including mRCC (n = 6), was conducted to specifically evaluate the efficacy and safety of everolimus in a Chinese population [26]. Results demonstrated that everolimus doses of 5 mg and 10 mg daily were well tolerated, and 67% of patients experienced stable disease as their best overall tumor response [26]. Median duration of everolimus exposure for patients with mRCC was 26.4 weeks (6.1 months). Herein, we report results of a larger study of everolimus in Chinese patients with mRCC.

## Methods

### Study design and treatment

In this open-label, multicenter phase 1b study, patients received everolimus 10 mg (2 × 5-mg tablets) daily until objective tumor progression (according to RECIST, version 1.0), unacceptable toxicity, death, or study discontinuation for any other reason. If a patient experienced unacceptable toxicity, dose reductions to 5 mg daily or 5 mg every other day or dose interruptions were permitted. This study was conducted according to the ethical principles of the Declaration of Helsinki. The study protocol was reviewed and approved by the Independent Ethics Committee or Institutional Review Board for each participating study center in China. Written informed consent was obtained from each patient before screening procedures were initiated.

Primary end points were safety and tolerability. Secondary end points included disease control rate (DCR, defined as the proportion of patients with a best overall tumor response of complete response [CR], partial response [PR], or stable disease [SD]), overall response rate (ORR: CR + PR), PFS, and overall survival (OS). Additional exploratory outcomes included the evaluation of systemic pre-dose everolimus exposure levels and of the relationship between pre-dose exposure and predefined safety/efficacy end points.

### Patients

Adult Chinese patients with mRCC who were intolerant to or who progressed while still on or after stopping treatment with VEGFr-TKI therapy within 6 months were enrolled (N = 64). Patients were required to have confirmed clear cell mRCC with at least 1 measurable lesion (RECIST, version 1.0), a Karnofsky Performance Status (KPS) ≥ 70%, and adequate bone marrow, liver, and renal function. Patients with brain metastases were eligible if they were neurologically stable and did not require corticosteroids. Patients were ineligible if they had received previous chemotherapy, immunotherapy, radiotherapy, or an investigational agent (at the time of study protocol preparation, pazopanib and axitinib were included) within 4 weeks of study entry or sunitinib and/or sorafenib within 2 weeks of first everolimus dose. Previous treatment with mTOR inhibitors was not permitted. Patients who had received chronic treatment with immunosuppressive agents were ineligible for the study, whereas low-dose corticosteroids were permitted. Patients with severe and/or uncontrolled medical conditions including unstable angina, congestive heart failure, uncontrolled hypercholesterolemia, or diabetes were ineligible.

### Assessments and statistical methods

Safety assessments included the occurrence of adverse events (AEs), serious AEs (SAEs), and monitoring of hematology, biochemistry, serum lipid profile, and vital signs. AE monitoring continued for 4 weeks after patients received their last dose of study drug. The safety population was defined as all patients who received ≥ 1 dose of everolimus and had ≥ 1 postbaseline safety assessment. The frequency distribution of patients with AEs and laboratory data abnormalities were summarized by worst CTC grade based on Common Terminology Criteria for Adverse Events (CTCAE) v 3.0 [27]. The estimated raw incidence (95% CI) of grade 3–4 AEs and of SAEs was identified as the primary safety analysis. Pulmonary events were diagnosed by the participating investigators and not confirmed independently. Kaplan-Meier estimates evaluated the time to onset of non-infectious pneumonitis.

For efficacy evaluation, CT/MRI scans and/or bone scan if bone metastases were present or suspected were carried out at baseline and every 8 weeks for the first year of treatment, then every 12 weeks and at treatment discontinuation. Assessment of overall lesion response was performed per RECIST criteria (version 1.0), and radiologic information was reviewed and evaluated by the investigator and/or local radiologists. The efficacy population comprised all patients who received ≥ 1 dose of everolimus. Efficacy end points included DCR, ORR, PFS, and OS. The Kaplan-Meier method was used to analyze time to event end points (PFS and OS). PFS was defined as the time from the start of treatment to the date of documented tumor progression or death due to any cause, whichever came first. In the absence of a PFS event, patients were censored at the time of their last valid tumor assessment. OS was defined as the time from treatment start to death due to any cause; patients lost to follow-up or who were still alive at analysis cut-off date were censored at their last contact date. The final data analysis cut-off date was November 30, 2011.

For PK assessments, pre-dose PK samples were collected on day 1 of cycles 2 and 4 by direct venipuncture into polypropylene tubes containing K2 ethylene diamine tetraacetic acid. Patients were then instructed to take everolimus after the pre-dose sample had been collected, so that an accurate trough level could be determined. Everolimus concentrations in whole blood were determined by a liquid chromatography tandem mass spectrometry method after a solid phase extraction. The lower limit of quantitation was 0.3 ng/mL. Pre-dose trough samples (C_min_) collected at cycle 2 day 1 and cycle 4 day 1 could represent the minimum everolimus exposure under steady-state conditions. A linear mixed model analysis of log-transformed steady-state pre-dose minimum plasma concentrations (C_min_) normalized to the dose of 10 mg/day, including the cycle as fixed effect and the patient as random effect, overall mean, 90% CI, and inter- and intra-patient variability, were estimated. Exploratory analyses of the relationship between log-transformed, time-normalized everolimus concentration and time to occurrence of pre-selected safety/efficacy end points were performed using the Cox regression model.

The sample size was identified as the minimum number of patients required to assess everolimus-related toxicities in the studied population and was not based on formal calculation. The study included a preplanned interim analysis of safety, which was conducted 4 months after treatment initiation by the last patient enrolled, and a final analysis after 12 months of treatment. All patients still receiving everolimus at the time of the final analysis were given the option to continue treatment until disease progression or unacceptable toxicity.

## Results

### Patient disposition

At the time of the final analysis, 54 patients (84%) had discontinued treatment. The most frequent reasons for discontinuation included disease progression (n = 26, 41%), AEs (n = 15, 23%), withdrawal of consent (n = 6, 9%), and death (n = 3, 5%).

### Demographics and baseline characteristics

The full analysis set comprised 64 patients who received ≥ 1 dose of everolimus 10 mg/day (Table 1). The majority of patients were male (n = 44, 69%) and all patients were of Chinese ethnicity. Median age was 52 years and most patients were < 65 years of age (n = 58, 91%). Most patients presented with histologically or cytologically confirmed clear-cell adenocarcinoma of the kidneys (n = 62, 97%) and the majority (n = 41, 64%) had a time since initial diagnosis ≥ 24 months. Twenty-six patients (41%) had metastatic involvement of ≥ 3 organs, and the lungs were the most common site of metastasis (n = 52, 81%). All patients had undergone surgery and received previous VEGFr-TKI therapy, with half the population receiving previous sorafenib.

**Table 1:** Patient demographics and baseline disease characteristics

**Characteristic**	**Everolimus 10 mg/day N = 64**
**Age, y, median (range)**	52 (19–75)
**Sex, n (%)**	
Female	20 (31)
Male	44 (69)
**Previous VEGFr-TKI therapy, n (%)**	
Sunitinib	21 (33)
Sorafenib	32 (50)
Axitinib	4 (6)
Pazopanib	7 (11)
**All previous systemic therapy, n (%)**	
Immunotherapy	29 (45)
Chemotherapy	6 (9)
Targeted therapy	64 (100)
Other	10 (16)
**Previous surgery, n (%)**	64 (100)
**Previous radiotherapy, n (%)**	17 (27)
**Disease sites, n (%)**	
1	9 (14)
2	29 (45)
≥3	26 (41)
**Common sites of metastasis, n (%)**	
Lung	52 (81)
Bone	22 (34)
Liver	11 (17)
Pleural effusion (malignant)	11 (17)
Retroperitoneal mass	9 (14)
Mediastinum	8 (13)
Thoracic lymph nodes	7 (11)

VEGFr-TKI, vascular endothelial growth factor receptor-tyrosine kinase inhibitor.

### Treatment exposure

Median duration of everolimus therapy was 4.1 months (range, 0.0-16.1 months). The mean cumulative dose was 1616.88 mg, with a mean dose intensity of 8.95 mg/day (median, 9.90 mg/day), corresponding to a mean relative dose intensity of 0.89 (median, 0.99). The majority of patients (n = 45, 70%) had a relative dose intensity between 0.90 and < 1.10. Overall, 37 patients (58%) required ≥ 1 dose reduction/interruption of everolimus: 14 patients (22%) had their dose reduced/interrupted once, and 23 patients (36%) required dose reductions/interruptions more than once over the course of the study. The most common reason for dose reduction/interruption was management of AEs (n = 24, 38%).

### Safety

Seven patients died within 28 days of treatment discontinuation. The principal cause of death was progressive disease in 5 patients; 1 patient died of respiratory failure suspected to be study drug-related, and 1 patient died of multi-organ failure not suspected to be study drug-related.

A total of 27 patients (42%, 95% CI, 30-55%) reported ≥ 1 on-treatment SAE. The most common SAEs occurring in ≥ 3% of patients included dyspnea (9%), pyrexia (6%), lung infection (3%), anemia (3%), multi-organ failure (3%), respiratory failure (3%), and renal failure (3%). SAEs were suspected to be treatment related in 11 patients (17%). Treatment-related SAEs occurring in ≥ 3% of patients were pyrexia (5%) and anemia (3%).

AEs regardless of relationship to study drug were reported in 62 patients (97%). The most frequently occurring AEs were anemia (64%), hypertriglyceridemia (55%), mouth ulceration (53%), hyperglycemia (52%), hypercholesterolemia (50%), pyrexia (41%), increased lactate dehydrogenase (38%), fatigue (31%), increased gamma-glutamyltransferase (GGT, 31%), and rash (31%) (Table 2). A total of 48 patients (75%, 95% CI, 63-85%) experienced at least one grade 3 or 4 AE regardless of relationship to study drug. Common grade 3/4 AEs were anemia (20%), hyperglycemia (13%), increased GGT (11%), hyponatremia (8%), dyspnea (8%), hypertriglyceridemia (6%), and lymphopenia (6%).

**Table 2:** All-grade AEs occurring in ≥ 10% of patients and corresponding grades 3 and 4 AEs

	**Everolimus 10 mg/day (n = 64)**		
**AE, n (%)**	**All-grade**	**Grade 3**	**Grade 4**
Anemia	41 (64)	10 (16)	3 (5)
Hypertriglyceridemia	35 (55)	4 (6)	0 (0)
Mouth ulceration	34 (53)	2 (3)	0 (0)
Hyperglycemia	33 (52)	8 (13)	0 (0)
Hypercholesterolemia	32 (50)	0 (0)	0 (0)
Pyrexia	26 (41)	1 (2)	0 (0)
Increased blood lactate dehydrogenase	24 (38)	1 (2)	1 (2)
Fatigue	20 (31)	1 (2)	0 (0)
Increased gamma-glutamyltransferase	20 (31)	7 (11)	0 (0)
Rash	20 (31)	0 (0)	0 (0)
Increased blood creatinine	19 (30)	0 (0)	1 (2)
Cough	18 (28)	0 (0)	0 (0)
Increased aspartate aminotransferase	17 (27)	0 (0)	0 (0)
Increased alanine aminotransferase	15 (23)	1 (2)	0 (0)
Epistaxis	15 (23)	1 (2)	0 (0)
Hypocalcemia	14 (22)	0 (0)	0 (0)
Interstitial lung disease	14 (22)	1 (2)	0 (0)
Leukopenia	14 (22)	0 (0)	0 (0)
Pruritus	14 (22)	0 (0)	0 (0)
Peripheral edema	13 (20)	2 (3)	0 (0)
Decreased platelet count	13 (20)	1 (2)	0 (0)
Diarrhea	12 (19)	1 (2)	0 (0)
Dyspnea	12 (19)	1 (2)	4 (6)
Increased blood alkaline phosphatase	11 (17)	1 (2)	0 (0)
Hypokalemia	11 (17)	0 (0)	3 (5)
Lymphopenia	10 (16)	4 (6)	0 (0)
Nasopharyngitis	9 (14)	0 (0)	0 (0)
Dizziness	8 (13)	0 (0)	0 (0)
Insomnia	8 (13)	0 (0)	0 (0)
Increased blood creatine phosphokinase	7 (11)	0 (0)	0 (0)
Decreased appetite	7 (11)	1 (2)	0 (0)
Decreased hemoglobin	7 (11)	2 (3)	0 (0)
Hyponatremia	7 (11)	5 (8)	0 (0)
Nausea	7 (11)	0 (0)	0 (0)
Upper respiratory tract infection	7 (11)	0 (0)	0 (0)
Increased white blood cell count	7 (11)	0 (0)	0 (0)

AE, adverse event.

Non-infectious pneumonitis events were reported in 20 patients (31%) and included interstitial lung disease (n = 14, 22%), pneumonitis (n = 5, 8%), and pulmonary fibrosis (n = 1, 2%). Grade 3 non-infectious pneumonitis was reported in 4 patients (6%), all of whom had lung metastases at study entry: 3 events improved to grade 1/2 after steroid therapy, oxygen inhalation, and/or dose adjustment; 1 event of pulmonary fibrosis improved to grade 2 following discontinuation of everolimus. Overall, no grade 4 non-infectious pneumonitis was reported. The probability of onset of non-infectious pneumonitis was estimated to be 6% (95% CI, 3-16%) at 1 month and 32% (95% CI, 21- 46%) at 4 months. The median time to first occurrence of non-infectious pneumonitis was not reached.

The most frequently observed laboratory abnormalities were decreased hemoglobin (80%), increased triglycerides (77%), increased fasting glucose (72%), increased cholesterol (61%), and decreased absolute lymphocyte count (61%). Common grade 3/4 laboratory abnormalities included decreased hemoglobin (23%), decreased absolute lymphocytes (20%), and increased GGT (16%).

### Efficacy

**Figure 1:**
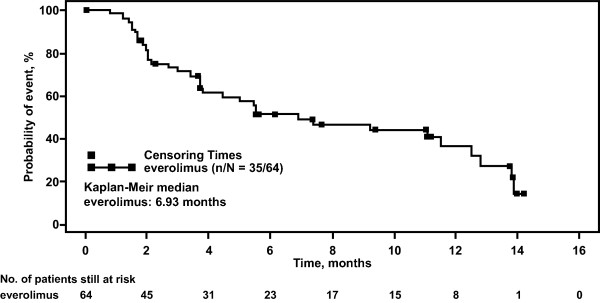
**Kaplan-Meier estimate of progression-free survival.** The plot depicts the probability of progression-free survival over time (months) for patients who received everolimus (full analysis set population). The square symbol represents censoring times. The number of patients still at risk is shown for each time point.

**Figure 2:**
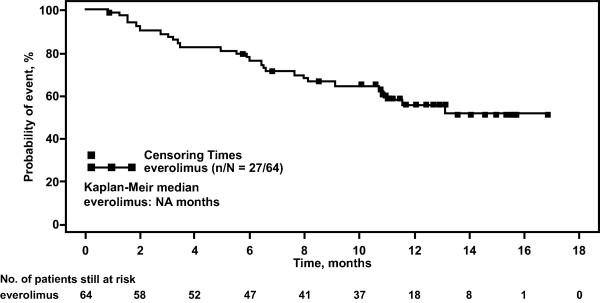
**Kaplan-Meier estimate of overall survival.** The plot depicts the probability of overall survival over time (months) for patients who received everolimus. The square symbol represents censoring times. The number of patients still at risk is shown for each time point.

At the data cut-off date, median PFS was estimated to be 6.9 months (95% CI, 3.7-12.5 months, Table 3 and Figure 1). The estimated probability of PFS was 62% (95% CI, 47-73%) at 4 months, 52% (95% CI, 38-64%) at 6 months, and 36% (95% CI, 22-51%) at 12 months. Median OS was not reached (Figure 2). The estimated probability of OS was 76% (95% CI, 64-85%) at 6 months and 56% (95% CI, 42-68%) at 12 months.

**Table 3:** Summary of efficacy measures

	**Everolimus 10 mg/day, N = 64**
**PFS**	
PFS events, n (%)	35 (55)
Progression	26 (41)
Death	9 (14)
Censored observations, n (%)	29 (45)
Median PFS, months (95% CI)	6.9 (3.7-12.5)
**Best Overall Response, n (%)**	
Complete response	0
Partial response	3 (5)
Stable disease	39 (61)
Progressive disease	8 (13)
Unknown*	14 (22)
**Response analysis, n (%)**	
ORR (CR or PR)	3 (5)
[95% CI for ORR]	[1-13]
DCR (CR or PR or SD)	42 (66)
[95% CI for DCR]	[53–77]
**OS**	
Median OS, months (95% CI)	NR (10.7-NR)
Probability of OS at 6 months,% (95% CI)	76 (64–85)
Probability of OS at 12 months,% (95% CI)	56 (42–68)

*Includes patients with no tumor assessments.

PFS, progression-free survival; ORR, overall response rate; OS, overall survival; CR, complete response; PR, partial response, SD, stable disease; NR, not reached.

Confirmed objective tumor responses (all PRs) evaluated by investigator assessment were seen in 3 patients, corresponding to an ORR of 5% (95% CI, 1-13%, Table 3). Stable disease was reported in 39 patients (61%). Everolimus therapy in this patient population was associated with a DCR of 66% (95% CI, 53-77%).

### Pharmacokinetics

Mean C_min_ after administration of everolimus 10 mg/day was 21.4 ± 12.4 ng/mL at cycle 2, day 1 (n = 36) and 15.0 ± 9.97 ng/mL at cycle 4, day 1 (n = 22). Mean C_min_ after administration of everolimus 5 mg/day was 0.7 ng/mL at cycle 2, day 1 (n = 1) and 8.8 ± 1.14 ng/mL at cycle 4, day 1 (n = 3). Mean C_min_ at cycle 4, day 1 at the 10-mg/day dose was approximately twice the mean C_min_ value at the 5-mg/day dose, which confirms a dose-proportional increase in pre-dose exposure of everolimus after daily administration. In the analysis of PFS, the estimated risk ratio of 0.67 suggested a trend toward longer PFS with higher time-normalized everolimus C_min_. However, the corresponding 95% CI (0.273-1.668) included unity, thereby precluding conclusion of any statistically significant relationship. There was no apparent difference between patients in the everolimus time-normalized C_min_ categories of < 10 ng/mL, 10–25 ng/mL, and > 25 ng/mL and all grades of non-infectious pneumonitis and stomatitis/oral mucositis.

## Discussion

This phase 1b study was planned and conducted to evaluate the safety and efficacy profile of everolimus in Chinese patients with mRCC after failure of VEGFr-TKI therapy. Overall safety findings from this study were consistent with those reported in the phase 3 RECORD-1 study [10,28] and with a phase 1 study conducted in Chinese patients with advanced solid tumors [26]. In RECORD-1, everolimus 10 mg daily was generally well tolerated in patients with mRCC whose disease had progressed despite previous VEGFr-TKI therapy. Common AEs reported in patients treated with everolimus included stomatitis, infections, asthenia, fatigue, and diarrhea; common laboratory abnormalities included decreased hemoglobin, increased glucose, and increased cholesterol and triglycerides; pneumonitis occurred in 14% of patients (grade 3, 4%; grade 4, 0%) [10,28]. A phase 1 study investigated everolimus 5 mg and 10 mg daily in Chinese patients with different tumor types (breast cancer, non-small cell lung cancer, RCC, and gastric cancer), results of which confirmed the safety of everolimus in Chinese patients. Most AEs were grade 1 or 2, and the most common (all grades) were hyperglycemia, fatigue, and anemia [26]. Based on clinical results, guidelines for the appropriate management of AEs related to everolimus treatment have been developed [29,30].

In this study, expected known class-effect toxicities related to mTOR inhibitor therapy were observed, including anemia, hypertriglyceridemia, hyperglycemia, hypercholesterolemia, pulmonary events, and stomatitis. Most of the AEs were grade 1/2 in severity. Although anemia occurred in 64% of the patients, only 16% and 5% of patients experienced grade 3 or 4 events, respectively. It is important to note that a large portion of the study population had abnormal hematologic values at baseline, including grades 1–2 decreased hemoglobin in 52% of patients. Many patients also had abnormal biochemistry values at baseline, including 44% with increased triglycerides (grades 1–2) and 28% with increased glucose (grades 1–2). Non-infectious pneumonitis events (interstitial lung disease, pneumonitis, and pulmonary fibrosis) occurred in 31% of patients in this study. Although this percentage was higher than in the overall RECORD-1 population based on blinded investigator assessment (14%) [10], it was similar to the incidence reported based on prospective, blinded independent review of CT scans from patients in the RECORD-1 trial in whom a diagnosis of clinical pneumonitis was not made but who experienced radiographic changes while receiving everolimus (38.9%) [31]. Additionally, our results were consistent with the incidence of noninfectious pneumonitis reported in the Japanese subpopulation of RECORD-1 (27%) [32]. In the current study, most pulmonary events were grade 1 or 2; only 6% of patients experienced a grade 3 pulmonary event. In the overall population of RECORD-1, 4% of patients experienced a grade 3 pulmonary event and there were no grade 4 events; in the Japanese subpopulation of RECORD-1 there were no grade 3 events [10,32].

Efficacy outcomes in this study are comparable to those from RECORD-1 [10]. The confirmed objective tumor response rate (all PRs) was 5% in this study versus 1.8% in RECORD-1 [10]. DCR was 66%, which was also comparable to the 69% of patients who achieved PR or SD in RECORD-1. In RECORD-1, everolimus 10 mg daily provided clinical benefit to patients with mRCC whose disease had progressed despite VEGFr-TKI therapy (median PFS, 4.9 months vs 1.9 months for placebo; HR 0.33, *P* < .001). In comparison, the median PFS associated with everolimus in Chinese patients refractory to previous VEGFr-TKI therapy in this single-arm study was 6.9 months.

## Conclusions

Everolimus provides clinical benefit to Chinese patients with mRCC, with comparable efficacy to that observed in Western patients. Everolimus was generally well tolerated and safety findings were consistent with those from RECORD-1 and from a phase 1 study of everolimus in Chinese patients with advanced solid tumors. This study provides further evidence supporting the use of everolimus as a standard of care in patients with VEGFr-TKI–refractory mRCC.

## Competing interests

Jun Guo, Yiran Huang, Xu Jhang, Fangjian Zhou, Yinhao Sun, Shukui Qin, and Zhangqun Ye declare they have no competing interests. Hui Wang, Patrick Straub, Nicoletta Pirotta, and Sven Gogov are employees of Novartis Pharmaceuticals, Inc. Annette Jappe is an employee of and holds stock in Novartis Pharmaceuticals, Inc.

## Authors’ contributions

Jun Guo, Yiran Huang, Xu Zhang, Fangjian Zhou, Yinghao Sun, Shukui Qin, and Zhangqun Ye were investigators for the study, gathered data, and participated in drafting the manuscript. Jun Guo was principal investigator and participated in the study design and results review. Hui Wang, Annette Jappe, Patrick Straub, Nicoletta Pirotta, and Sven Gogov participated in study design, data analysis, and drafting the manuscript. All authors read and approved the final manuscript.

## Pre-publication history

The pre-publication history for this paper can be accessed here:

http://www.biomedcentral.com/1471-2407/13/136/prepub
